# Supplementary figures and images for: Goniothalamin Induces Necroptosis and Anoikis in Human Invasive Breast Cancer MDA-MB-231 Cells
Source: Int J Mol Sci. 2019 Aug 14;20(16):3953. doi: 10.3390/ijms20163953 (PMC6720804; doi:10.3390/ijms20163953)

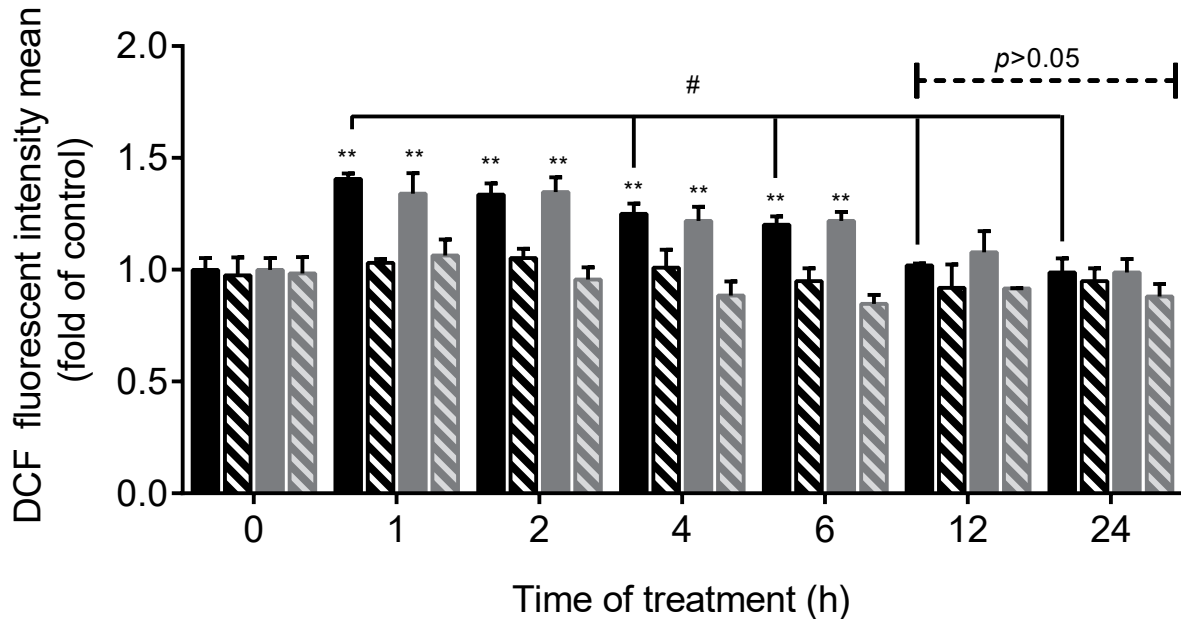

Supplement: Supplementary file 1 [file ijms-20-03953-s001.pdf]
